# Investigation of a subunit protein vaccine for HFRS based on a consensus sequence between envelope glycoproteins of HTNV and SEOV

**DOI:** 10.1016/j.virusres.2023.199149

**Published:** 2023-06-20

**Authors:** Rongrong Liu, Yunhua Lv, Wenjie Sun, Min Li, Ningning Ge, Cheng Zhu, Yaxin Ding, Ziyu Liu, Ruixue Ma, Yuxiao Huang, Shiyuan Hou, Qikang Ying, Tianle Gu, Fang Wang, Lingling Nie, Youchun Wang, Weijin Huang, Jiayi Shu, Xingan Wu

**Affiliations:** a Department of Microbiology, School of Basic Medicine, Fourth Military Medical University, Xi'an, China; b Northwest University, Xi'an, China; c Clinical Center for Biotherapy, Zhongshan Hospital & Zhongshan Hospital (Xiamen), Fudan University, Shanghai, China; d Institute Pasteur of Shanghai, Chinese Academy of Sciences, Shanghai, China; e Tianjin Key Laboratory of Function and Application of Biological Macromolecular Structures, School of Life Sciences, Tianjin University, Tianjin, China; f Division of HIV/AIDS and Sex-transmitted Virus Vaccines, Institute for Biological Product Control, National Institutes for Food and Drug Control (NIFDC) and WHO Collaborating Center for Standardization and Evaluation of Biologicals, Beijing, China

**Keywords:** Subunit vaccine, Consensus sequence, HFRS, HTNV, SEOV

## Abstract

Due to the global resurgence of hemorrhagic fever with renal syndrome (HFRS), more attention is being focused on this dangerous illness. In China and Korea, the only vaccines available are the virus-inactivated vaccine against Hantaan virus (HTNV) or Seoul virus (SEOV), but their efficacy and safety are inadequate. Therefore, it is important to develop new vaccines that are safer and more efficient to neutralize and regulate areas with a high prevalence of HFRS. We employed bioinformatics methods to design a recombinant protein vaccine based on conserved regions of protein consensus sequences in HTNV and SEOV membranes. The S2 Drosophila expression system was utilized to enhance protein expression, solubility and immunogenicity. After the Gn and Gc proteins of HTNV and SEOV were successfully expressed, mice were immunized, and the humoral immunity, cellular immunity, and *in vivo* protection of the HFRS universal subunit vaccine were systematically evaluated in mouse models. These results indicated that the HFRS subunit vaccine generated elevated levels of binding and neutralizing antibodies, particularly IgG1, compared to that of the traditional inactivated HFRS vaccine. Additionally, the spleen cells of immunized mice secreted IFN-r and IL-4 cytokines effectively. Moreover, the HTNV-Gc protein vaccine successfully protected suckling mice from HTNV infection and stimulated GC responses. In this research, a new scientific approach is investigated to develop a universal HFRS subunit protein vaccine that is capable of producing effective humoral and cellular immunity in mice. The results suggest that this vaccine could be a promising candidate for preventing HFRS in humans.

## Highlights

• By utilizing bioinformatics techniques, we have identified conserved regions within the m genes of HTNV and SEOV. These regions have been utilized to design a universal subunit protein vaccine for HFRS.
• The transmembrane domain of Gn and Gc in the envelope glycoproteins of hantaan and seoul viruses was eliminated to enhance the solubility of protein and augment the level of vaccine expression.
• The S2 drosophila expression system was utilized to acquire the universal subunit vaccines for hantaan and seoul viruses.
• The neutralization induced by HTNV-Gc protein exhibited a greater protective effect compared to inactivated vaccines, as observed both *in vitro* and *in vivo*.

## 1 Introduction

Hemorrhagic fever with renal syndrome (HFRS) is a natural focal disease caused by Hantavirus infection that is prevalent in 38 countries and regions in Asia and Europe (Zheng et al., 2018; Zou and Sun, 2020). Annually, approximately 20,000 cases of hantavirus-related diseases occur worldwide (Jiang et al., 2017), with HTNV and SEOV being the primary hantavirus species in China. Severe cases of HFRS caused by the HTNV virus have a mortality rate of 5−10%, while SEOV can cause moderate disease with a mortality rate of 1−2% (Avšič-Županc et al., 2019; Liu et al., 2019). Recently, the HFRS epidemic has resurged in some areas of China, such as Chang'an District, Shaanxi Province. Furthermore, Hantavirus is a potential biological warfare agent that has been included in a verification list by the international Biological Weapons Convention. Unfortunately, no effective therapeutic drugs or prophylactic vaccines are available for clinical use; therefore, finding effective ways to address HFRS is essential for ensuring national biosecurity and people's well-being.

During virus maturation, the envelope glycoprotein (GP) of hantavirus is further cleaved into two proteins, Gn and Gc. These proteins are essential for immune cell recognition (Li et al., 2016) and consist of an N-terminal extracellular domain, a hydrophobic transmembrane sequence (TM), and a C-terminal cytoplasmic sequence (Hepojoki et al., 2012; Sankar et al., 2017). Studies have found that HTNV Gn and Gc can activate B lymphocytes to produce specific antibodies against HTNV; these antibodies are mainly neutralizing antibodies, which can effectively protect HTNV-infected animals or patients with HFRS (Hooper et al., 2001; Spik et al., 2008). HTNV glycoprotein (GP) has been identified as a vital antigen protein in HFRS vaccine research because it can potentially induce neutralizing antibodies and protective cellular immune responses. Ma et al. identified 79 novel 15-mer T-cell epitopes on the HTNV GP, of which 20 peptides were dominant target epitopes (Ma et al., 2015). Therefore, GP may be capable of inducing humoral immunity and promoting the clearance of the intracellular virus (Li et al., 2016). Regarding antigen sites, there are nine neutralizing antigen sites, two hemagglutination antigen sites on Gn, and seven neutralizing antigen sites on Gc (Arikawa et al., 1989).

Epidemiological and clinical efficacy evaluations of HFRS vaccines have revealed that the whole virus-inactivated vaccine induces low titers of neutralizing antibodies and inadequate cellular immunity, and its protective effect is limited (Zhang et al., 2007). Vaccination may not provide complete protection against the virus (Ma et al., 2016). Moreover, the preparation of monovalent and bivalent vaccines involves the culturing, inactivation, purification, and mixing of the live virus, which is a major safety issue (Dong et al., 2005; Hjelle, 2002).

The development of novel genetically engineered vaccines for HFRS has become a viable alternative to inactivated vaccines, such as the hantavirus nucleic acid vaccine, viral vector vaccine, and virus-like particle (VLP) vaccine, some of which have already been tested in clinical studies. Hooper et al. demonstrated that a recombinant DNA vaccine containing the HTNV M gene administered via a gene gun could confer sterile protection against HTNV, SEOV, and DOBV in hamsters (Hooper et al., 2001). Cheng et al. engineered chimeric HTNV-VLP using defective CHO and baculovirus, in which GM-CSF or CD40L is attached via a GPI anchor. These chimeric HTNV VLP induced strong humoral and cellular immune responses and protected HTNV in C57BL/6 mice (Cheng et al., 2016). Despite progress in developing HFRS genetically engineered vaccines, several challenges remain, such as low expression of target proteins and immune response. Therefore, further innovation and optimization of hantavirus genetic engineering vaccines are necessary to make them suitable for practical use.

Subunit vaccines are safe and easy to manufacture, and multiple components can be combined to provide simultaneous inoculation (Liang et al., 2018; Qu et al., 2018; Sun et al., 2017a). George F. Gao's team has developed multiple recombinant subunit vaccines in response to the COVID-19 pandemic; one of these vaccines, ZF2001, has been given conditional marketing authorization in China and authorization for emergency use in Uzbekistan, Indonesia, and Colombia (Yang et al., 2021). Xu et al. designed a chimeric recombinant subunit vaccine based on the RBD dimer, which can protect against various SARS-CoV-2 strains (Xu et al., 2022).

Using bioinformatics, Yang et al. created a recombinant subunit vaccine containing residues 319–545 of the spike protein of SARS-CoV-2 (Yang et al., 2020). In addition, Jin et al. developed a single protein-based tetravalent DENV vaccine that can induce immunity against a wide range of dengue virus genotypes (3177 strains) (Sun et al., 2017a). Additionally, a Zika subunit recombinant protein vaccine was designed using the same method and was shown to provide protective immunity in a mouse challenge model and a 100% protective effect in the rhesus monkey protection test (Liang et al., 2018). Different hantavirus types exhibit high homology and conservation, which has become an advantage in designing universal vaccines (Sun et al., 2020). Sankar et al. used a bioinformatics approach to T-cell epitopes to design a broad-spectrum polypeptide subunit vaccine against five HFRS-related pathogens (Sankar et al., 2017). However, this study was only at the antigen design stage, and no experimental data has verified its immune protection effect. These studies suggest that the HFRS subunit vaccine has potential clinical application value.

In this study, a bioinformatics approach was employed to design a recombinant subunit protein vaccine for hemorrhagic fever with renal syndrome (HFRS), which was based on the known envelope glycoprotein gene sequences of HTNV and SEOV viruses from the NCBI database. The Drosophila S2 expression system expressed the HFRS recombinant subunit protein component. The immunogenicity, cellular immunity, and *in vivo* protection of the HFRS universal subunit vaccine were systematically evaluated in mouse models. Furthermore, the immunological mechanism of the vaccine was investigated, and germinal center formation in mice immunized with the HFRS universal subunit vaccine was explored. It was found that the HFRS universal subunit vaccine induced effective humoral immunity, cellular immune response, and *in vivo* protection in mouse models, with an increased number of GC B and Tfh cells in the lymph nodes of immunized mice. In conclusion, the HFRS universal subunit protein vaccine that was designed using bioinformatics exhibits good immunogenicity and protective effects *in vivo*. In addition, the vaccine is expected to become a HFRS preventive vaccine that is safe, stable, protective, and suitable for large-scale production.

## 2 Materials and methods

### 2.1 Animals

SPF-grade BALB/c adult female mice aged 6–8 weeks and newborn suckling mice aged 0–3 days were purchased from the Animal Research Center of the Fourth Military Medical University in Xi'an, China. The animal study protocols were approved by the Committee of Laboratory Experimentation of the Fourth Military Medical University Animal Center (No. 20,210,403). All possible steps were taken to minimize animal suffering and to reduce the number of animals used in the experiment.

### 2.2 Viruses and cells

Our laboratory provided HTNV 76–118 strains, while Professor Weijin Huang provided VSVΔG*-HTNVG and VSVΔG*-SEOVG pseudoviruses from the National Institute of Food and Drug Control (NIFDC). For the cell neutralization experiments, Vero E6 cells (Vero C1008, ATCC#CRL 1586) were cultivated in DMEM (Gibco, Grand Island, NY, USA) with 10% fetal bovine serum (BBI, China). Drosophila S2 cells (ATCC, USA) were also utilized.

### 2.3 Vaccine design

To obtain two sequences representing envelope genes of circulating Hantaan virus (HTNV) and Seoul virus (SEOV), we downloaded all envelope gene sequences from the NCBI database and filtered out duplicates. To avoid bias towards the larger group of HTNV, consensus envelope sequence (>50% consensus) of each serotype were calculated individually first, second rated amino acids were chosen to fill the gap if the sequence consistence was lower than 50%. HTNV and SEOV Gn head and Gc protein sequences were then obtained based on their consensus sequences. Detailed methods were previously published (Sun et al., 2017b).

### 2.4 Expression and purification of recombinant proteins

The codon preference of Drosophila S2 cells was utilized to optimize and synthesize two envelope glycoprotein consensus sequences, namely, the Hantaan virus and Seoul virus. A C-terminal thrombin cleavage site was then introduced into the Gn head region (1062 bp) and Gc extracellular domain (1347 bp) of both viruses. Subsequently, these genes were inserted into the S2 cell expression vector pMT/Bip/V5-His-A. Six recombinant plasmid transfection reagents were transfected into Drosophila S2 cells, and 25 μg/ml blasticidin was added to screen for positive cells. The expression of the target protein was induced by adding 10 μM CdCl_2_ to the culture. The cells and the culture supernatant were collected and incubated with an anti-histidine primary antibody, followed by an alkaline phosphatase (AP)-labeled secondary antibody for Western blot analysis. The expression and secretion of the target protein were then detected by BCIP/NBT substrate reaction.

The constructed S2 cell line, which expresses the recombinant protein, was grown and cultured in serum-free medium containing 10 μg/ml blasticidin to maintain selection pressure. When the cell concentration reached 1 × 10^7 cells/ml, 10 μM CdCl_2_ was added to induce protein expression. The cell supernatant was harvested after 6 days of suspension culture in a 28 °C incubator, centrifuged, and concentrated by filtration. The concentrated supernatant was then incubated with nickel-chelated agarose (Ni-NTA Agarose) at 4 °C with rotation for 4 h to separate the unbound proteins. After elution and three rounds of PBS dialysis, the recombinant target protein with a histidine tag was purified. The purified protein was analyzed by SDS‒PAGE and BCA protein quantification (Pierce Biotechnology, IL, USA) to evaluate the yield and purity.

### 2.5 Mouse immunization

The experiment was conducted with 6 groups of 5 mice each. The mice were immunized subcutaneously at two points with 10 μg/mouse of purified Hantan HTNV-Gn, Hantan HTNV-Gc, Seoul SEOV-Gn, and Seoul SEOV-Gc proteins, as well as HFRS-inactivated vaccine and PBS as controls, with aluminum hydroxide (Invivogen, USA) as an adjuvant. After the third immunization, the mice were sacrificed 7–10 days later, blood was collected for antibody detection, splenocytes were isolated for Elispot and flow cytometry, and inguinal lymphocytes were isolated for flow cytometry.

### 2.6 Enzyme-linked immunosorbent assay

Polystyrene microplates coated with Gn and Gc proteins of HTNV and SEOV were incubated overnight at 4 °C. Next, PBST containing 1% BSA was added and incubated at 37 °C for 1 h. Serial dilutions of serum samples (experimental wells) and naïve mouse serum samples (negative control) were then added and incubated at 37 °C for 1 h. HRP-goat anti-mouse IgG (Zhuang Zhi Bio, China) was added to each well and incubated at 37 °C for 1 h, followed by the addition of TMB (3,3′,5,5′-tetramethylbenzidine) substrate (TIANGEN, China). The reaction was stopped by adding ELISA stop solution (Solarbio, China) after standing at room temperature for 10 min. The absorbance value at 450 nm of each well was measured, and a P/N value greater than 2.1 was considered positive. The reciprocal of the highest dilution of the serum considered positive was used to calculate the specific antibody titers of HTNV and SEOV based on the geometric mean titer (GMT) of particular antibodies in each group of immunized mouse serum.

The Gn and Gc proteins of HTNV and SEOV were coated similarly to identify different types of immunoglobulins. Mouse serum samples were diluted, and HRP-labeled IgG was used as IgG1, IgG2a, IgG2b, IgA and IgM (Southern Biotech, USA). The absorbance at 450 nm was then measured to detect the quantity of each immunoglobulin isotype.

### 2.7 Pseudovirus neutralization assay

The mouse immune sera were inactivated by heating at 56 °C for 30 min, serially diluted with PBS and incubated with pseudovirus (VSVΔG*-HTNVG) at 37 °C for 1 h. Subsequently, Vero E6 cells were infected with the serum pseudovirus mixture and incubated at 37 °C for 24 h. The relative fluorescence intensity (RLA) of each well was measured using a PerkinElmer kit. The serum inhibition was calculated by the following formula: (RLA of virus control - RLA in test sample)/RLA of virus control. The serum neutralizing titer was determined as the 50% maximum inhibitory dilution (ID_50_), and the neutralizing antibody titer of each mouse was calculated using the Reed-Muench method.

### 2.8 Live HTNV neutralization assay

The neutralizing ability of antibodies was evaluated using a sandwich ELISA method. Vero E6 cells were cultured in 96-well plates until they reached 60% confluence. The plates were then preincubated with serial dilutions of mouse serum (1:80, 1:100, 1:200, 1:400, 1:800, and 1:1600) and HTNV (1 × 10^5 TCID_50_) at 37 °C for 1 h. Next, the virus-serum mixture was removed, 0.2 ml of overlay medium was added, and the plates were incubated at 37 °C with 5% CO_2_ for 10 days. The plates were subjected to three freeze‒thaw cycles at -80 °C and RT, and the supernatant was collected for ELISA. The capture antibody used was HTNV NP monoclonal antibody, and HRP-1A8, an HRP-conjugated HTNV NP monoclonal antibody, was used as the detecting antibody. Neutralizing antibody titers were determined as the maximum serum dilution in which three of four quadruplicated wells did not show positive HTNV NP.

### 2.9 Enzyme-linked immunospot (ELISPOT) assay

Experiments were conducted according to a protocol in the IFN-γ and IL-4 Elispot kits (Mabtech, NackaStrand, Sweden). The mouse spleen was removed and gently ground with a 200 μm cell stainer, followed by the addition of RBC lysing buffer to remove red blood cells. The spleen cells were resuspended in PRMI-1640 medium with 10% FBS and added to the coated Elispot plates at a concentration of 3 × 10^5 cells/well. The corresponding stimuli were added to the plate, including recombinant antigen protein (1 μg/well), positive control concanavalin A (ConA) (1 μg/well), and negative control PBS. The plate was incubated at 37 °C for 36–48 h. After the cell liquid was discarded, the dish was washed five times with PBS, followed by the addition of detection antibodies against IFN-γ and IL-4. Streptavidin-horseradish peroxidase (HRP) was added as the secondary antibody, and TMB substrate was added to terminate the reaction until prominent spots appeared. After drying in the shade, the number of spots was read using a cellular immune spot analyzer, and the number of spot-forming cells was counted.

### 2.10 Suckling mouse protection experiment

Inactivated immune sera were blended with a lethal dose of HTNV virus at a 1:1 ratio and incubated at 37 °C for 1 hour. Subsequently, BALB/c suckling mice were intraperitoneally injected with 100 μl of the serum virus mixture per mouse, with 10 mice per group. The morbidity and mortality of the suckling mice were monitored for 14 days.

### 2.11 Flow cytometry (FACS)

Isolated inguinal lymph nodes and spleens from immunized mice were ground with a 200 μm cell stainer and resuspended in flow wash solution (2% FBS in PBS) on ice to maintain cell viability and prepare single-cell suspensions. Mouse anti-CD16/CD32 monoclonal antibody (mAb) was added to the single-cell suspensions to block Fc before staining. Flow cytometry was then used to analyze the cells according to standard procedures. The antibodies used to stain mouse immune cells were APC—CD19^+^, APC/Cyanine7-CD38^−^, PE-CD95^+^, FITC—CD3^+^, APC—CD4^+^, PE-PD1^+^, APC/Cyanine7-CXCR5^+^, APC—CD19^+^, FITC—CD44^+^ and PE/Cyanine7-CD138^+^. Data acquisition and analysis were conducted using Novo Express software.

### 2.12 Immunofluorescence staining and microscopy

The inguinal lymph nodes of immunized mice were isolated and fixed with 4% paraformaldehyde. Subsequently, paraffin sections were created and blocked with 3% BSA at room temperature for 30 min. The primary antibody (GL7) was then added and incubated at 4 °C overnight. After the sections were washed with PBS three times, secondary antibodies (goat anti-rat antibody labeled by HRP) were added and incubated at room temperature for 50 min. Following three washes with PBS, 647-TSA was added and stored in the dark for 10 min at room temperature. After three TBST washes, the sections were repaired by microwave, and staining with the other three primary antibodies (IgD, CD21, CD3) was conducted in succession according to the same method. Images were collected by scanning the sections with a scanner (Pannoramic) and analyzed by Case Viewer software.

### 2.13 Statistical analysis

Statistical analysis was conducted using Prism (GraphPad Software 9.2.0). Student's t-test was employed to evaluate P values for comparing two groups, while one-way ANOVA was used to assess statistically significant differences for more than two groups. Statistical significance was established when P values were < 0.05 (*p* < 0.05 (*), *p* < 0.01 (**), *p* < 0.001 (***), and *p* < 0.0001 (****)).

## 3 Results

### 3.1 Designing and developing a universal subunit vaccine for HFRS

Sequence alignment analysis of 166 Hantaan virus envelope glycoprotein sequences (1135 amino acids) and 98 Seoul virus envelope glycoprotein sequences (1133 amino acids) from the NCBI database yielded a consensus sequence of the HTNV envelope glycoprotein and a common sequence of the SEOV envelope glycoprotein, in which amino acid residues with a frequency of over 50% at each site were chosen as the candidate sequences. The sequence alignments were developed with WebLogo Weblogo (https://weblogo.berkeley.edu/logo.cgi) (Fig. S4b and c). With the exception of G354 and M359 in the HTNV Gn region, which were highly mutated, analyzed by a sequence logo generator Weblogo 3 (Fig. S4a), at 35% and 45% consensus separately, and were marked with different-sized amino acids indicated consistence.

**Fig. 1:**
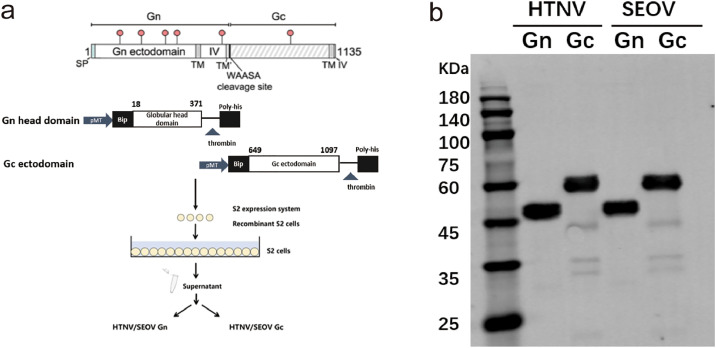
Design strategy and construction of a universal subunit vaccine for HFRS. (a) Construction of recombinant plasmid and expression of recombinant protein. The N-terminus of the target protein was the sequence of the Bip signal peptide from S2 cells, and the C-terminus was the thrombin cleavage site and histidine tag. The recombinant protein was expressed using the Drosophila eukaryotic expression system. (b) Western blot analysis of recombinant protein. The expected size without oligosaccharide sites of the Hantaan virus recombinant protein: Gn head region (43.1 KDa), Gc ectodomain region (53.8 KDa); The expected size without oligosaccharide sites of Seoul virus recombinant protein: Gn head region (43.2 KDa), Gc ectodomain region (53.6 KDa).

Analysis of amino acid sequence evolutionary alignment results indicated that the two common sequences were located in the middle region of the dense distribution of published sequences of the same virus and exhibited a close evolutionary distance with multiple sequences. We then optimized the two consensus envelope glycoprotein sequences according to the codon bias of Drosophila S2 cells, removing the retention signal to improve protein expression, solubility, and immunogenicity. Fig. 1a outlines the process of expressing HTNV and SEOV Gn and Gc recombinant proteins using the Drosophila S2 cell line. To begin, the consensus sequence of Hantan virus and Seoul viral envelope glycoprotein was used to obtain the Gn head region (1062 bp) and Gc outer membrane region (1347 bp) gene fragments of HTNV and SEOV, respectively, which were then linked to the Drosophila S2 cell expression vector to construct the recombinant expression plasmid. This plasmid was then transfected into Drosophila S2 cells, and after selection, induction, and identification, a S2 cell line with stable expression of the recombinant protein was obtained. Finally, the S2 cell line stably expressing the recombinant protein was expanded and induced to express and purify the Gn and Gc recombinant proteins of HTNV and SEOV, respectively (Fig. 1a). Finally, we successfully obtained a total of 4 recombinant proteins: the Gn head region of HTNV (HTNV-Gn), the Gc ectodomain region (HTNV-Gc), the Gn head region of SEOV (SEOV-Gn), and the Gc ectodomain region (SEOV-Gc) (Fig. 1b).

### 3.2 The HFRS universal subunit vaccine can elicit specific binding and neutralizing antibodies

**Fig. 2:**
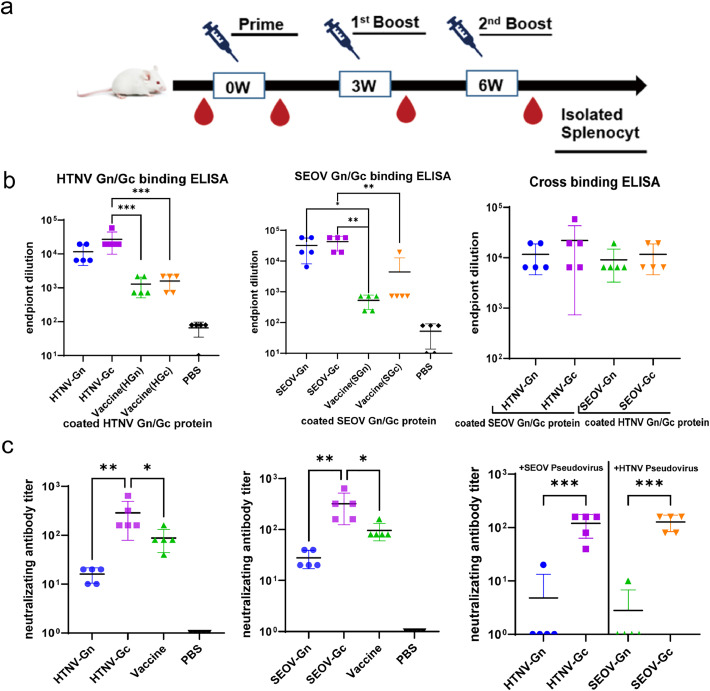
Humoral immune responses to the HFRS universal subunit vaccine. Mice were administered three doses of HFRS universal subunit vaccine plus alum adjuvant, with five mice in each group. As a control, HFRS inactivated vaccine (Vaccine) and PBS were also used. On Day 7 after the third vaccination, serum was collected for antibody titer determination. (a) Vaccination schedule. (b) The titers of Gn and Gc protein-specific IgG in immune serum (detected by end-point dilution ELISA, *n* = 5). (c) The neutralizing antibody (nAb) titers of immune serum detected by VSVΔG*-HTNVG and VSVΔG*-SEOVG pseudovirus neutralization assays. Each symbol shows the antibody titer of an individual mouse.

BALB/c mice were immunized with four proteins, HTNV-Gn, HTNV-Gc, SEOV-Gn, and SEOV-Gc, and an inactivated HFRS vaccine (referred to as the vaccine group) and PBS, with aluminum hydroxide as an adjuvant. After three immunizations, serum was collected (Fig. 2a) to detect the immunogenicity of specific binding antibodies and neutralizing antibodies induced by the proteins.

ELISA was employed to detect the specific binding antibodies of Gn and Gc in mouse serum. The results showed that the HTNV-Gc group exhibited the highest geometric mean titer (GMT) of specific binding antibodies, reaching 1:27,216, while the HTNV-Gn group exhibited a titer of 11,664 (Fig. 2b). There was no remarkable difference between the two groups. On the other hand, the HFRS vaccine group exhibited lower antibody levels, with titers of 1:1296 and 1:1584 for HTNV Gn and Gc, respectively (Fig. 2b).

Research revealed that antibodies to SEOV Gn and Gc were present in the SEOV-Gn, SEOV-Gc, and inactivated HFRS vaccine-immunized groups. The SEOV-Gc group showed the highest specific binding antibody GMT (Fig. 2b). The above results indicate that high levels of specific binding antibodies may be produced in mice immunized with HTNV-Gn, HTNV-Gc, SEOV-Gn, and SEOV-Gc protein. Moreover, the HTNV- and SEOV-immunized groups possessed cross-binding antibodies (Fig. 2b).

The results of the pseudovirus neutralization test indicated that the highest level of neutralizing antibodies was induced by the HTNV-Gc group in mice, with a GMT of 1:288. However, the neutralizing antibody GMT of the HTNV-Gn group was significantly lower at 1:16. The level of neutralizing antibody induced by the HFRS-inactivated vaccine was also found to be lower, with a GMT of 1:88. Similarly, the SEOV-Gc group was observed to have a higher neutralizing antibody GMT than that of the SEOV-Gn group and the HFRS inactivated vaccine group (Fig. 2c). These results demonstrate that the Gc protein of both HTNV and SEOV can effectively stimulate the production of neutralizing antibodies in mice, and the efficacy of this method is significantly better than that of inactivated vaccines. Additionally, the HTNV-Gc and SEOV-Gc groups were observed to produce high-titer cross-neutralizing antibodies (Fig. 2c).

Neutralizing antibodies in the serum of HTNV-Gn- and HTNV-Gc-immunized mice were detected by a live HTNV microneutralization assay, with results that were in agreement with those of the sham virus method. HTNV-Gc-immunized mice had high titers of neutralizing antibodies (Fig. S1).

### 3.3 Analysis of the Ig subtypes in immune sera from individuals receiving the HFRS universal subunit vaccine

**Fig. 3:**
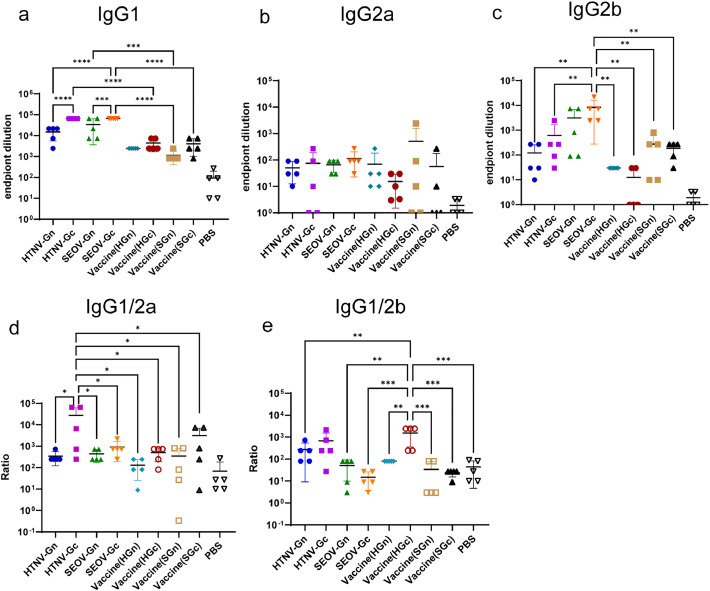
Distribution of Ig subtypes in immune sera from the HFRS universal subunit vaccine. The antibody subtypes in immune sera with the HFRS universal subunit vaccine were classified by measuring the titers of IgG1, IgG2a, and IgG2b with end-point dilution ELISA (*n* = 5). (a) The IgG1 titer in immune sera. (b) The IgG2a titer in immune sera. (c) The IgG2b titer in immune sera. (d) The IgG1/IgG2a titer ratios were calculated (*n* = 5). (e) The IgG1/IgG2b titer ratios were also calculated (*n* = 5).

Through ELISA, we determined the specific binding antibody subtypes in mouse immune sera, which included IgG1, IgG2a, IgG2b (Fig. 3a–c), IgA, and IgM (Fig. S2a and S2b). IgG1 had the highest titer among these immune sera, followed by IgG2a, IgG2b, and IgM. Additionally, the IgG1 titers of the HFRS inactivated vaccine group and the PBS group were significantly lower than that of the HFRS universal subunit vaccine group.

Th2 cells are T cells that aid B lymphocytes in producing antibodies. In mice, Th2-polarized responses are linked to IgG1 production, while Th1-polarized responses are associated with IgG2-type antibodies (Reinhardt et al., 2009; Stevens et al., 1988). To further clarify the Tfh differentiation results, we calculated the IgG1/IgG2a and IgG1/IgG2b ratios for each immunization group (Fig. 3d and e). The results indicated that the HFRS subunit vaccine mainly induced Th2-type cellular immune responses.

### 3.4 The T-cell immune response to the HFRS universal subunit vaccine

**Fig. 4:**
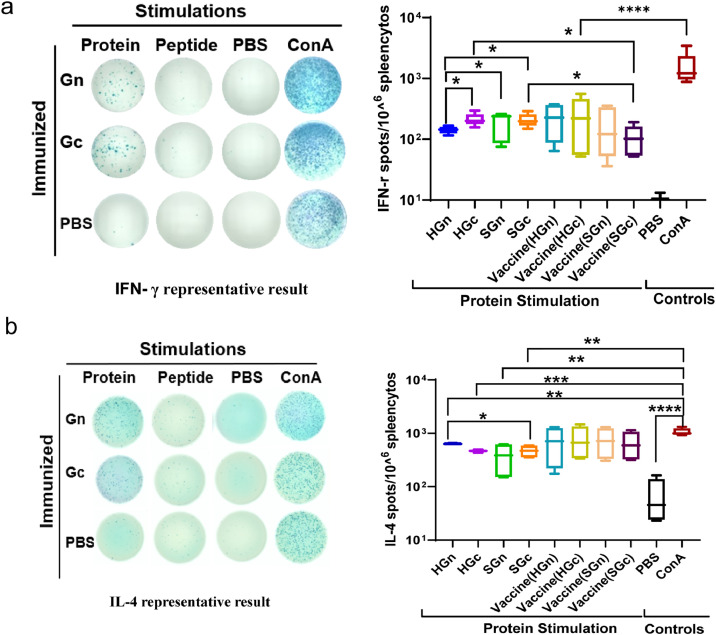
T-cell responses induced by the HFRS universal subunit vaccine. Splenocytes from immunized mice were stimulated with recombinant proteins of HTNV and SEOV. (a) The Th1 responses induced by the HFRS universal subunit vaccine were detected by IFN-γ Elispot assays. Representative results of the Elispot method and data statistics (*n* = 5). (b) The Th2 responses induced by the HFRS universal subunit vaccine were detected by IL-4 Elispot assays. Representative results of the Elispot method and data statistics (*n* = 5). Stimulants included recombinant proteins, peptides, negative control PBS and positive control ConA.

An ELISpot assay was used to assess the T-cell response induced by the HFRS universal subunit vaccine. The results showed that compared to splenocytes of mice the PBS control group, splenocytes of the mice in each immunized group secreted both IFN-γ and IL-4 cytokines, with no statistically significant difference between the groups (Fig. 4a and b). Notably, a higher level of IL-4 was observed than IFN-γ, indicative of a more robust Th2-type cell response, as evidenced by the higher IgG1 levels in the antibody typing response.

### 3.5 The HFRS universal subunit vaccine immune serum effectively provided adequate protection to suckling mice against HTNV lethal challenge

**Fig. 5:**
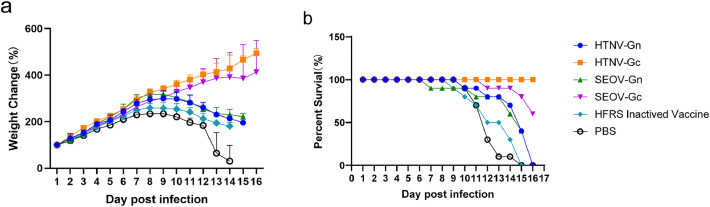
Protective capacity of the HFRS universal subunit vaccine in suckling mice *in vivo*. The HFRS universal subunit vaccine immune serum of each group was mixed with a lethal dose of HTNV and injected intraperitoneally into 10 suckling mice. The body weights of the mice were monitored and recorded daily for 16 days.(a) The changes in body weight were examined. (b) The survival rate of the suckling mice was determined.

The efficacy of the HFRS universal subunit vaccine was further evaluated by testing the passive protective effect of immune serum in suckling mice. The results showed that the body weight of the HTNV-Gc group increased steadily after the challenge, and the survival rate was 100% (Fig. 5a and b). In contrast, some mice in the SEOV-Gc group began to lose weight on the 11th to 15th day after the challenge, and the survival rate was 60% (Fig. 5a and b). The body weights of the other immunized groups began to decrease on the 9th day and then rapidly declined, and all the mice died within 16 days (Fig. 5a and b). These findings indicate that HTNV-Gc immune serum could provide adequate protection against HTNV challenge *in vivo*, and SEOV-Gc immune serum could provide certain cross-protection against HTNV challenge in suckling mice. However, HFRS whole virus inactivated vaccine immune serum could not protect the sucking mice from the HTNV challenge.

### 3.6 The HFRS universal subunit vaccine can stimulate a strong germinal center response in the lymph nodes

**Fig. 6:**
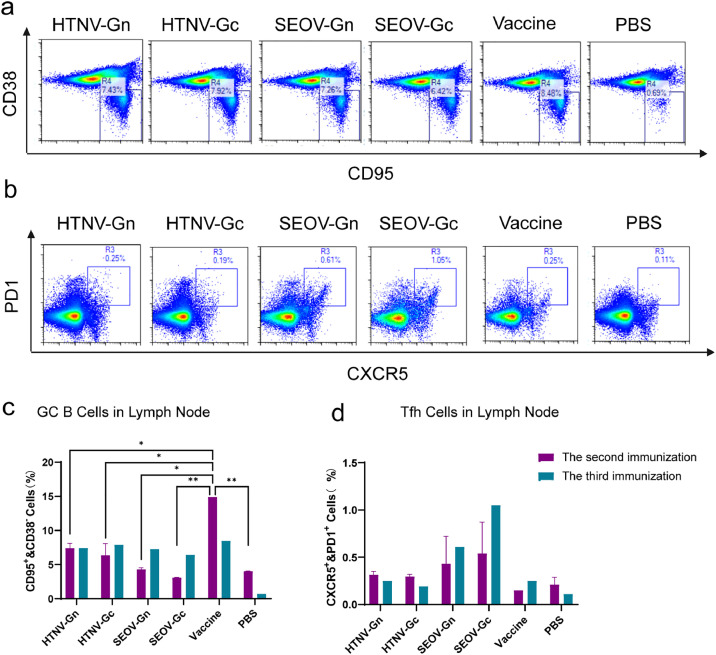
The universal subunit vaccine for HFRS triggers a germinal center response. Flow cytometry was used to measure the GC response in inguinal lymph nodes of immunized mice on Day 7 after the second immunization and Day 7 after the third immunization. This included the analysis of (a) GC B cells and (b) Tfh cells. (c, d) The percentage of each cell type in the lymph nodes of each immunized group.

Flow cytometry was used to detect germinal center (GC) B cells and T follicular helper (Tfh) cells in draining lymph nodes (LNs) and spleen seven days after the second and third immunizations. The results showed that inactivated HTNV-Gn, HTNV-Gc, SEOV-Gn, SEOV-Gc, and HFRS vaccines could stimulate large-scale GC B cells in LNs (Fig. 6a). Compared to the second immunization, after the third immunization, the GC B-cell secretion increased (Fig. 6c). However, the number of GC B cells in the spleen was much lower than that in the LNs (Fig. S3a and S3c). Similarly, Tfh cells could mature and differentiate after immunization with the HFRS universal subunit vaccine (Fig. 6b). Furthermore, GC B and Tfh cells were secreted less in the spleen than in LNs.

**Fig. 7:**
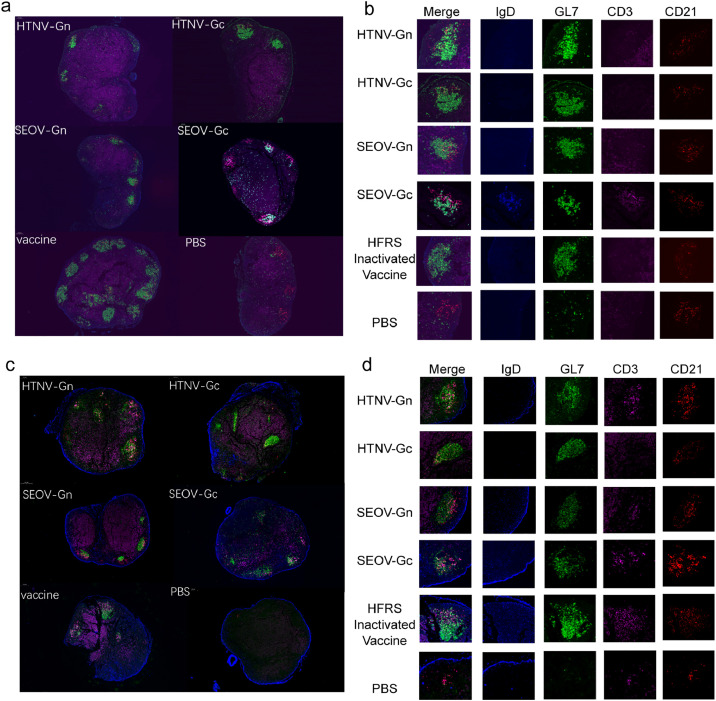
The universal subunit vaccine for HFRS is capable of inducing a germinal center response. Inguinal lymph nodes were collected for immunofluorescence staining of germinal centers. (a) On Day 7 after the second immunization, monoclonal antibodies against IgD (blue), GL7 (green), CD3 (rose), and CD21 (red) were used for immunofluorescence. The merged signal and each channel individually can be seen in the right local magnification selection of B cells. (b) The same procedure was conducted on Day 7 after the third immunization.

Immunofluorescence staining of tissue from lymph nodes was conducted to demonstrate that immunization by the HFRS universal subunit vaccine promotes germinal center (GC) formation. GCs are a population of GL7^+^ cells surrounded by follicular dendritic cells (fDCs, CD21^hi^) and IgD^+^ naïve B cells (Heesters et al., 2014) (De Silva and Klein, 2015). The results showed that the HFRS universal subunit vaccine could effectively induce GC formation regardless of the number of immunizations, while naïve and PBS mice could not (Fig. 7a–d). In the lymph nodes of mice immunized with the HFRS universal subunit vaccine, GL7^+^ cells were highly colocalized with fDCs (CD21^hi^) and differentiated from CD3^+^ T cells, indicating that GCs formed.

## 4 Discussion

Subunit protein vaccines offer a distinct advantage over traditional inactivated vaccines, as they can be designed to elicit a specific immune response through the use of bioinformatics. This design method considers various factors, such as structure, host-protein interactions, and pharmacokinetics. Furthermore, these vaccines are relatively safe, as the antigenic proteins do not potentially cause infection (Graham et al., 2019). Several universal influenza and HIV protein vaccines have already been tested in clinical trials, with the quadrivalent influenza virus subunit vaccine being approved by the Chinese State Food and Drug Administration. This vaccine is notable for its efficacy, purity, safety, and low rate of common adverse reactions.

In this study, we employed the Drosophila S2 cell expression system (Liang et al., 2018) to develop a subunit protein vaccine for HFRS. We optimized the antigen by considering the structural characteristics of the conserved regions of HTNV and SEOV, expressing the Gn and Gc protein components in segments, removing the retention signal of the gene transmembrane domain, and increasing the solubility of the expressed protein. We used eukaryotic expression system for the S2 expression to improve antigen folding and glycosylation modification, thereby further enhancing immunogenicity (Liang et al., 2018). The recombinant proteins were majorly N-linked glycoproteins (Shi and Elliott, 2004), confirmed with deglycosylation assay with PNGase F (NEB, UK) and Endo H (NEB, UK) and Western Blot. However, no publication indicated the type of glycosylation on SEOV envelope proteins, with the same assay, SEOV Gn glycoprotein might be also N-linked, though, SEOV Gc glycoprotein might be with other type of glycosylation modification.

Currently, the developmentand research of the hantavirus subunit vaccine mainly focus on the nuclear protein (NP) and glycoprotein (GP). Yoshimatsu et al. utilized a baculovirus expression vector system (BEVS) to express HTNV NP and GP, which showed strong antigenicity. After immunizing mice, the collected sera andspleen cells from mice were injected into suckling mice, demonstrating a partial protective effect against fatal HTNV challenge, despite deficient protein expression (Yoshimatsu et al., 1993). Razanskiene et al. (2004) reported that yeast cells could produce NPs of PUUV, DOBV, and HTNV stably and with high purity. GP is responsible for virus entry and can induce neutralizing antibodies to promote protective immune responses (Arikawa et al., 1992; Gavrilovskaya et al., 1998). NP has multiple T-cell epitopes and strong cellular immunogenicity (Yoshimatsu et al., 1996), but its ability to induce neutralizing antibodies is low. Therefore, research on the hantavirus GP subunit vaccine is of great importance.

In this study, we evaluated the humoral and cellular immune responses, as well as the protective effects of the HFRS subunit protein vaccine, in mouse models.The results indicated that the Gn and Gc of HTNV and SEOV could induce binding antibodies, and the Gc protein induced potent neutralizing antibodies. Moreover, the Gc protein-induced neutralizing antibodies of HTNV and SEOV exhibited good cross-reactivity and a broad spectrum. This was further demonstrated in the *in vivo* protection experiment of suckling mice, in which the HTNV-Gc protein vaccine was significantly more effective than the inactivated vaccine.

Studies have demonstrated that germinal center B cells, Tfh cells, and plasma cells are essential for forming effective neutralizing antibodies (nAbs) (Havenar-Daughton et al., 2017; Lederer et al., 2020). Katlyn Lederer et al. found that mice immunized with the SARS-CoV-2 mRNA vaccine exhibited a significant GC response, which resulted in neutralization of the SARS-CoV-2 virus *in vitro* (Lederer et al., 2020). Our research also showed that the universal HFRS vaccine and inactivated vaccine could induce GC responses. Immunofluorescence staining of the lymph nodes of immunized mice further revealed that both the HFRS universal subunit vaccine and inactivated vaccine could generate germinal center B cells, Tfh cells, and plasma cells.

The research revealed that the splenocytes of mice immunized with the HFRS subunit vaccine could produce IFN-γ and IL-4, with the latter being produced at a much higher magnitude. This indicates that the subunit vaccine may induce a Th2-biased Th cell response. This observation further confirmed that the HFRS subunit vaccine induced a much higher titer of IgG1 antibodies than those of IgG2a and IgG2b. This demonstrates that IL-4 is secreted after immunization, which causes most Th0 cells to differentiate into Th2 cells and promote humoral immunity.

Due to the fact that Hantavirus typically causes asymptomatic infection in adult BALB/c mice (Klingström et al., 2006), but can cause fatal neurological diseases or persistent infections in lactating and newborn mice (Araki et al., 2003; Yoo et al., 1993), we initially used the method of injecting immune serum into suckling mice and then attacking them to observe the protective effect through changes in body weight and survival rate. Furthermore, we conducted experiments in the later stage where BALB/c was first immunized and then challenged. Since BALB/c had no obvious pathological characteristics after infection with Hantavirus, we measured the Hantavirus load in the blood of mice from day 1 to day 7 after the challenge. qRT-PCR results showed that HTNV nucleic acid load levels in the Vaccine and PBS groups were similar and increased with time, without significant difference. In contrast, mice immunized with HTNV Gc and SEOV Gc had a low and static viral load, with significant differences between HTNV Gc and Vaccine groups, and between SEOV Gc and Vaccine groups (Fig. S5). These results indicate that HTNV-Gc or SEOV-Gc immunization can effectively protect mice against HTNV infection.

For more than a century, aluminum salts (alum) have been utilized to augment the immune system through billions of doses of vaccines (McKee and Marrack, 2017). Only a few adjuvants have been approved for human use, and alum is the most widely used (Clements and Griffiths, 2002). This is due to its low reactivity and cost-effectiveness (O'Hagan et al., 2017). In this study, alum was employed as an immune adjuvant. However, it has been observed that alum mainly induces Th2 cell responses rather than Th1 or cytotoxic T lymphocyte (CTL) responses (Marrack et al., 2009; Petrovsky and Aguilar, 2004), resulting in limitedantiviral and antitumor effects. Apart from alum, CpG, AS04, AS03, and MF59 are the only authorized vaccine adjuvants, yet the types and range of vaccines permitted are limited. Recently, Zhang et al. discovered that a colloidal manganese salt (Mn Jelly, MnJ) exhibited universal adjuvant activity, inducing humoral and cellular immune responses, particularly CTL activation. When administered intranasally, MnJ also acted as a mucosal adjuvant, causing high levels of IgA secretion. MnJ demonstrated impressive adjuvant effects on all tested antigens, even T-cell-independent antigens, indicating its potential as a promising adjuvant candidate (Zhang et al., 2021).

The incorporation of heat shock protein 70 (HSP70) as a fusion protein into the Dengue E80 subunit vaccine has been found to significantly enhance the E80-induced neutralizing antibody response (which was 20-fold higher than that of the alum adjuvant) (Chiang et al., 2016). Cheng et al. (2014) reported that using HSP70C-containing recombinant adenovirus RAD-GnS0.7-PCA-HSP70C expression vectors generated superior humoral and cellular immune responses compared to those of other Hantaan recombinant adenovirus (RAD-GnS0.7-PCAG and rAd-GnS0.7) vaccines. Consequently, to attain an improved immune response and address the low immunogenicity of Gn, future research should explore the possibility of utilizing alternative adjuvants to alum in the subunit vaccine.

In summary, our research has yielded a novel universal vaccine strategy to address the health prevention and control needs of HFRS infectious diseases. By using bioinformatics and structural biology of viral surface proteins, we designed and constructed HFRS universal subunit protein vaccines. These Gn and Gc protein vaccines of HTNV and SEOV have been evaluated for their immunogenicity and have been found to induce strong humoral and cellular immunity. The protective effect of Gc protein-induced neutralization was observed to be higher than that of inactivated vaccines *in vitro* and *in vivo*. This study provides a vaccine against HFRS with greater protection and safety than that of previous HRFS vaccines.

## Author statement

The authors confirm the availability of all data presented in the manuscript.

## Declaration of Competing Interest

The authors declare that they have no known competing financial interests or personal relationships that could have appeared to influence the work reported in this paper.

## Appendix Supplementary materials

Image, image 1

Image, image 2

Image, image 3

Image, image 4

Image, image 5
