# Supplementary figures and images for: Investigation of a subunit protein vaccine for HFRS based on a consensus sequence between envelope glycoproteins of HTNV and SEOV
Source: Virus Res. 2023 Jun 20;334:199149. doi: 10.1016/j.virusres.2023.199149 (PMC10410520; doi:10.1016/j.virusres.2023.199149)

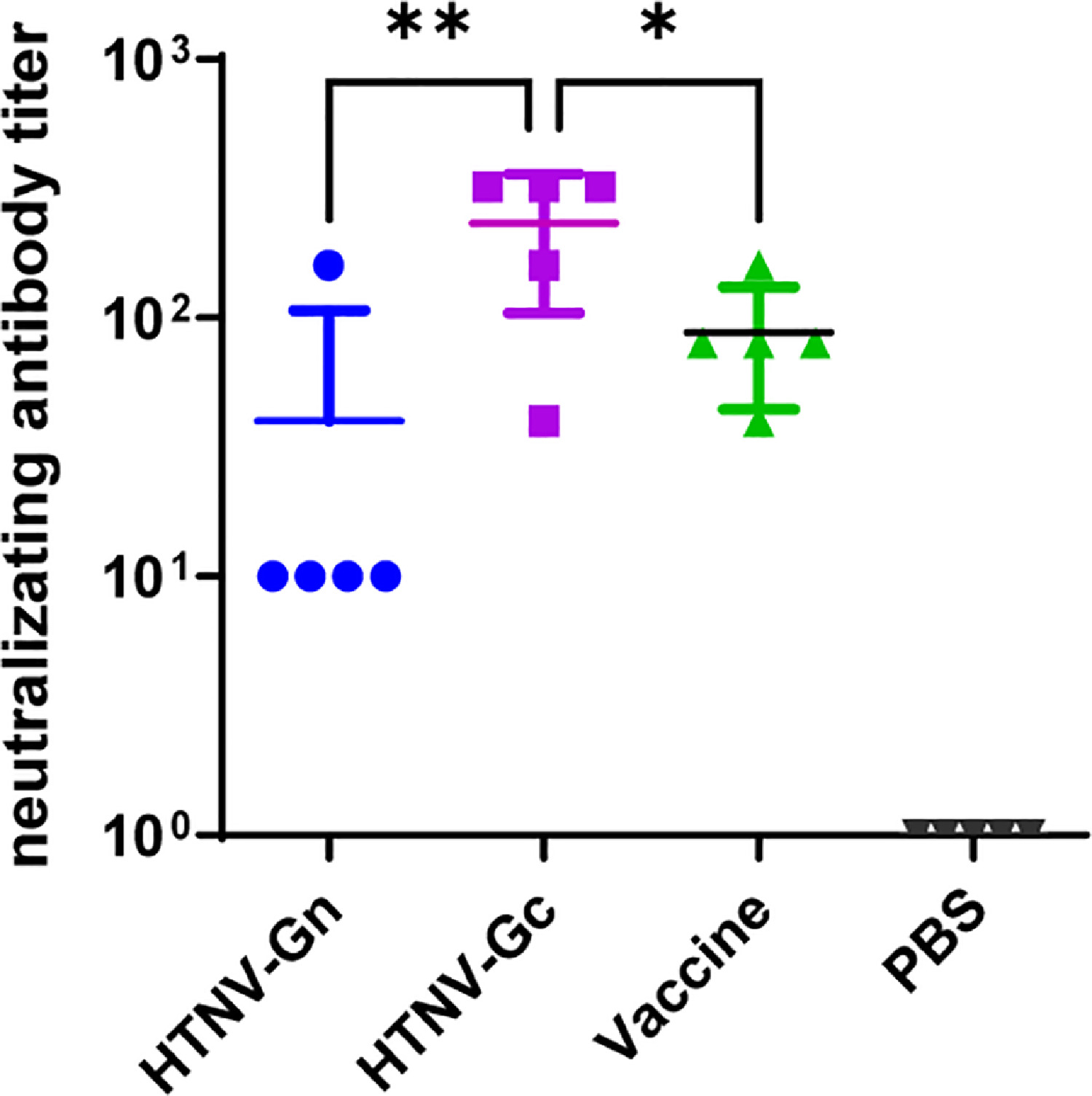

Supplement: Supplementary file 1 [file mmc1.jpg]

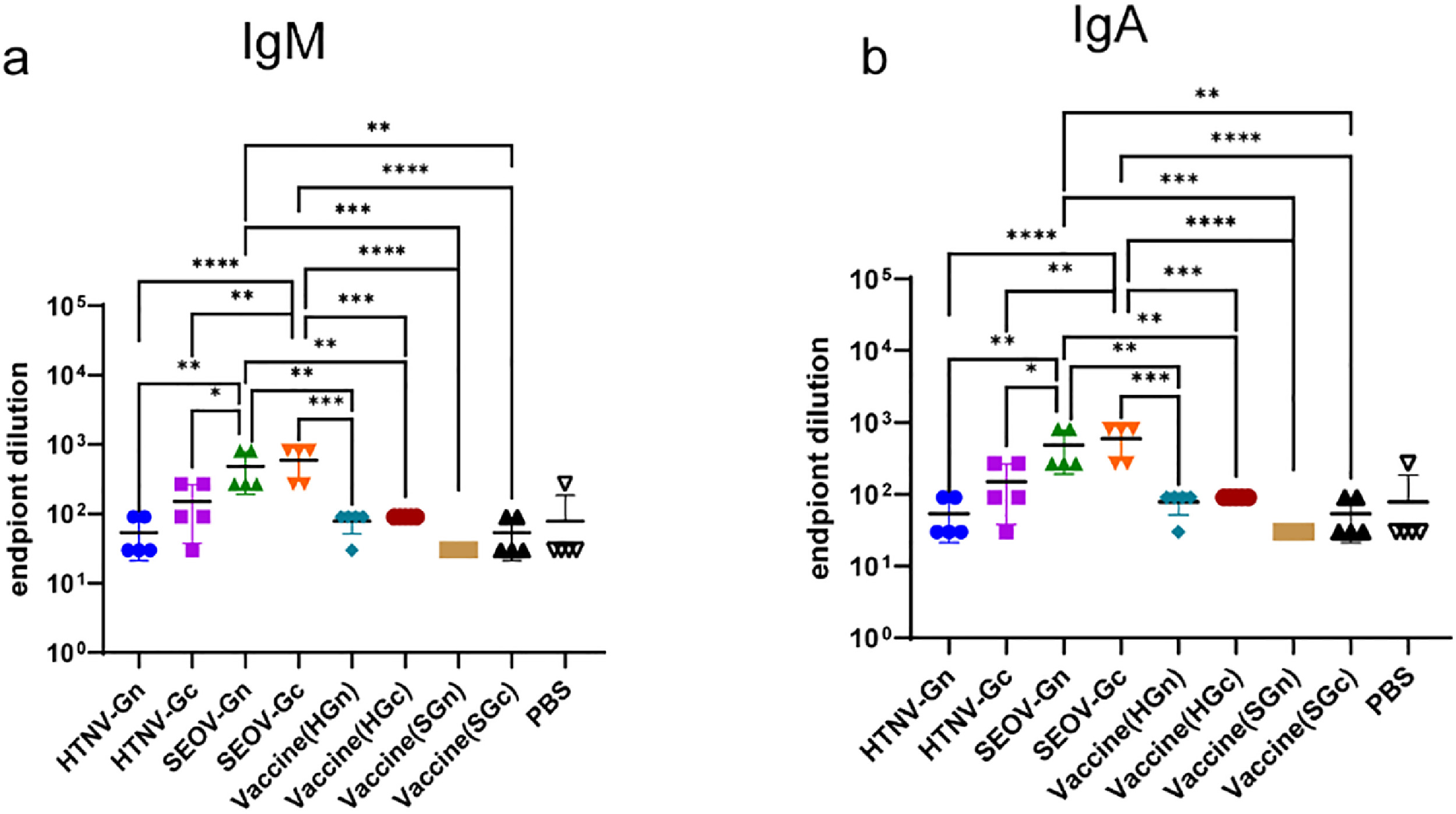

Supplement: Supplementary file 2 [file mmc2.jpg]

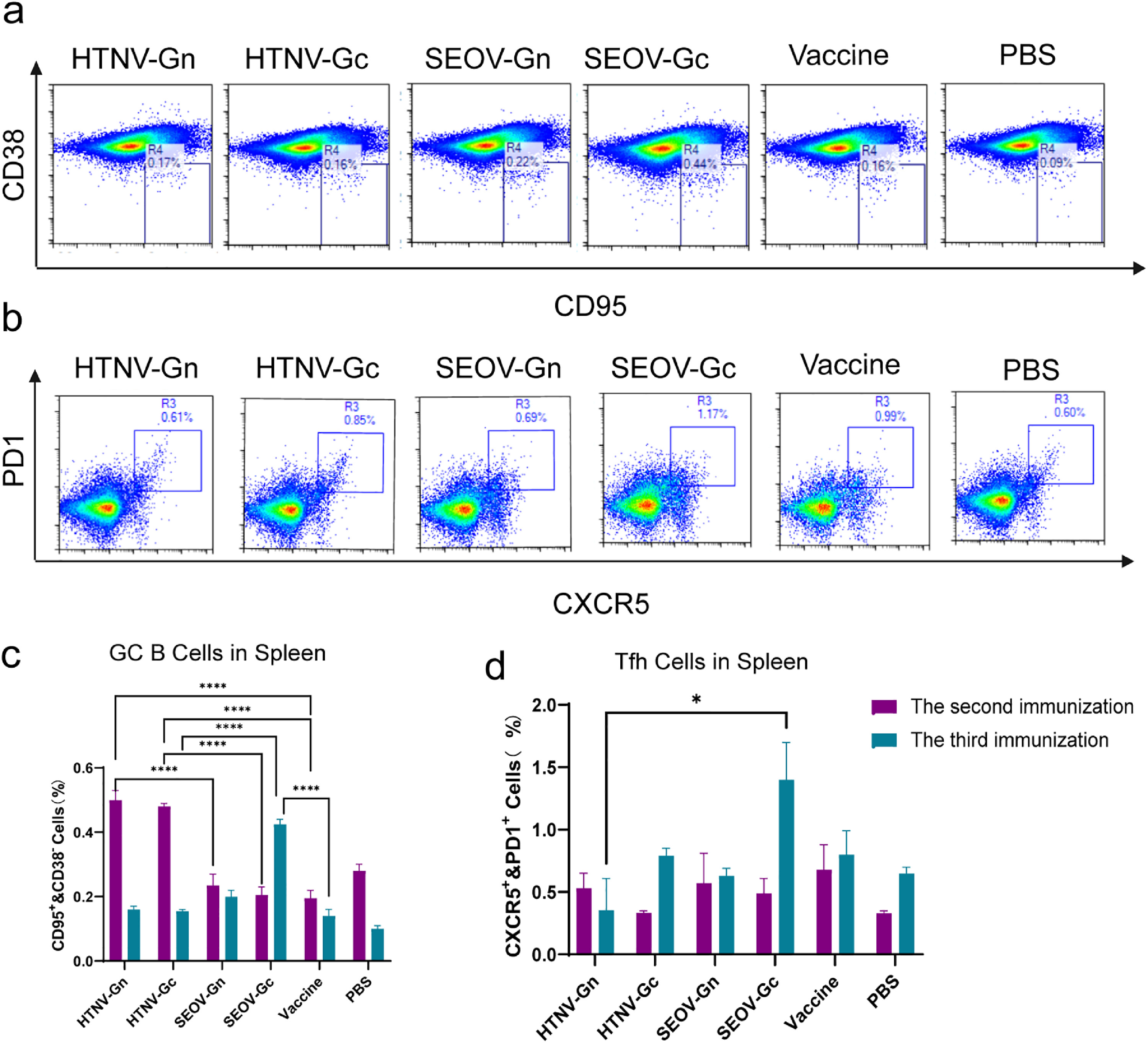

Supplement: Supplementary file 3 [file mmc3.jpg]

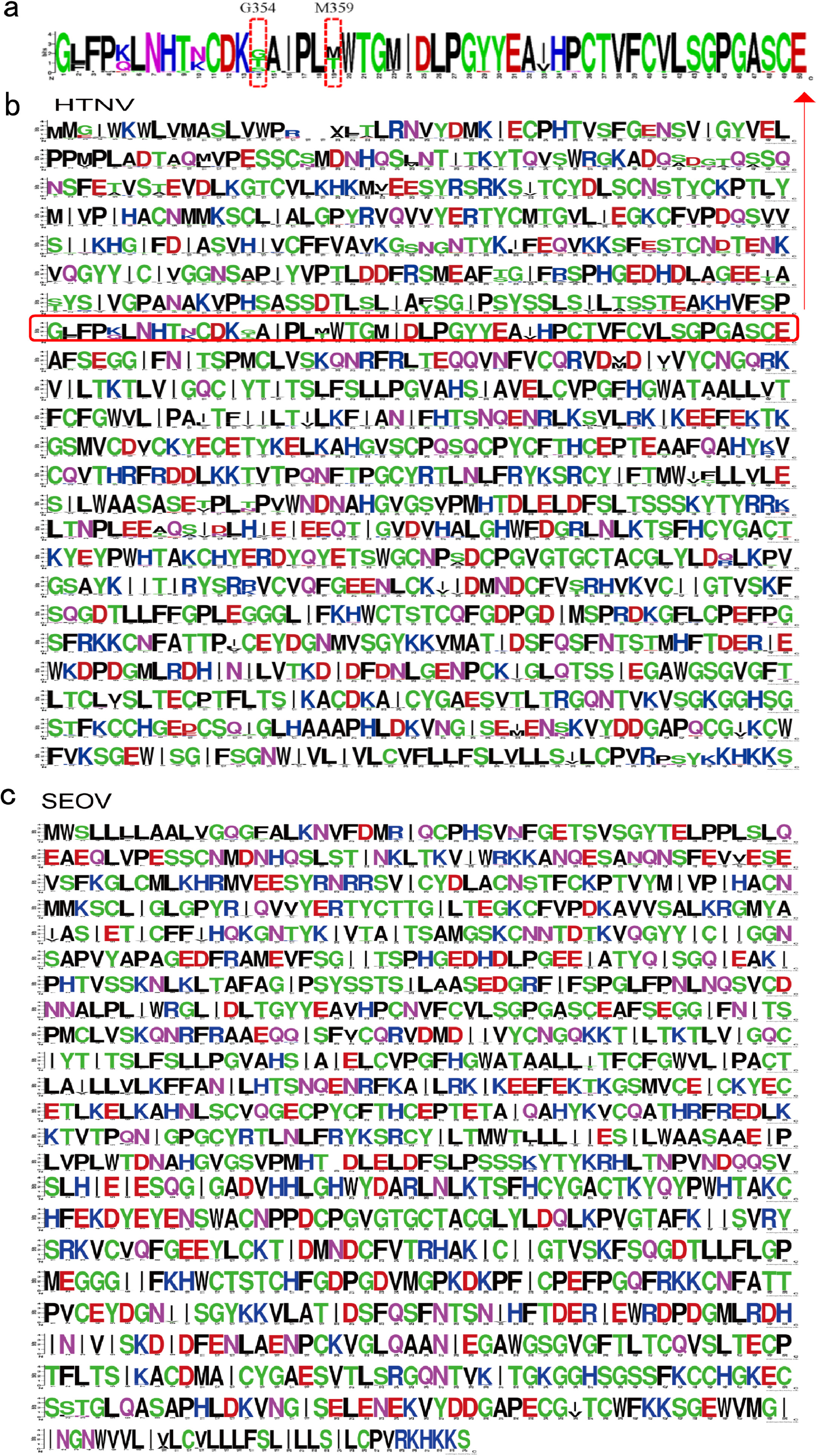

Supplement: Supplementary file 4 [file mmc4.jpg]

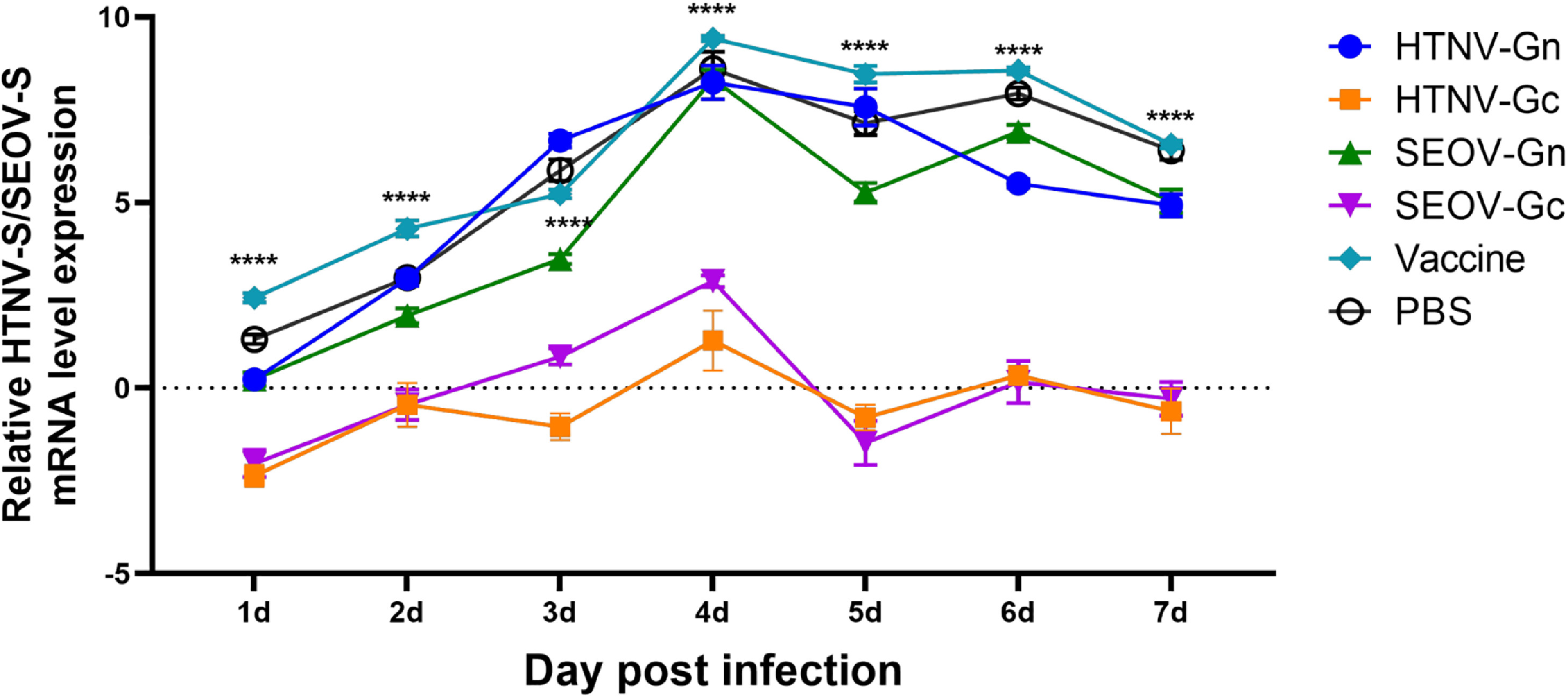

Supplement: Supplementary file 5 [file mmc5.jpg]
